# Bridging Additive Manufacturing and Electronics Printing in the Age of AI

**DOI:** 10.3390/nano15110843

**Published:** 2025-05-31

**Authors:** Jihua Chen, Yue Yuan, Qianshu Wang, Hanyu Wang, Rigoberto C. Advincula

**Affiliations:** 1 Center for Nanophase Materials Sciences, Oak Ridge National Laboratory, Oak Ridge, TN 37830, USA; yuany@ornl.gov (Y.Y.); wangh5@ornl.gov (H.W.); 2 ICSPI, 280 Joseph St, Kitchener, ON N2G 4Z5, Canada; qianshu@icspicorp.com; 3 Chemical Engineering, University of Tennessee at Knoxville, Knoxville, TN 37996, USA

**Keywords:** additive manufacturing, printing electronics, energy storage, sensors, AI, machine learning

## Abstract

Printing techniques have been instrumental in developing flexible and stretchable electronics, including organic light-emitting diode displays, organic thin film transistor arrays, electronic skins, organic electrochemical transistors for biosensors and neuromorphic computing, as well as flexible solar cells with low-cost processes such as inkjet printing, ultrasonic nozzle, roll-to-roll coating. The rise of additive manufacturing provides even more opportunities to print electronics in automated and customizable ways. In this work, we will review the current technologies of printing electronics (including printed batteries, supercapacitors, fuel cells, and sensors), especially with 3D printing. In this age of ongoing AI revolution, the application of AI algorithms is discussed in terms of combining them with 3D printing and electronics printing for a future with automated optimization, sustainable design, and customizable and scalable manufacturing.

## 1. Introduction

### 1.1. Printing Electronics

Since 1959, electronics printing has been traditionally responsible for manufacturing integrated circuits via photolithography, and in recent decades, it has also been an emerging technique used in fabricating flexible devices [1,2]. These flexible devices range from thin film displays, transistor arrays, solar cells, and sensors to neuromorphic computing [1,2]. Instead of relying on expensive high-vacuum, high-temperature processes of traditional semiconductor and electronics industries, recently developed electronics printing utilizes alternative, low-temperature, low-cost processes, including tape casting, inkjet printing, roll-to-roll manufacturing, or spray with ultrasonic nozzle [1,2].

### 1.2. Additive Manufacturing of Electronics

The current trend of 3D printing provides additional opportunities in printing electronics [3,4]. Compared to traditional subtractive manufacturing, it provides unprecedented flexibility for personalization, prototyping, and cost control, offering reduced reliance on supply chains and toolings [3,4]. This also leads to recent developments in 3D printing whole-structures such as energy storage devices, which is typically harder to achieve with traditional printing techniques [3,4]. The designer freedom and manufacturing cost of conventional battery manufacturing processes can be significantly enhanced by printing [2]. In conventional battery manufacturing, a typical streamline consists of (1). slurry preparation, (2). tape casting and drying, (3). calendaring, (4). electrode cutting, (5). packaging, and (6). electrolyte filling, while in 2D or 3D battery printing, the steps are now changed to: (1) electrode/electrolyte pattern design, (2) filament or ink preparation, (3) pre-printing treatment, (4) printing, (5) post-printing treatments, (6) packaging [2]. Pre-printing treatments may include the optimization of digital 3D models using computer-aided design (CAD) software for proper dimensions and tolerances design, drying moisture-sensitive filaments to prevent defects, as well as temperature and ventilation controls [5]. Post-printing treatments, on the other hand, are applied after printing to enhance mechanical properties, surface, and functionality with processes such as washing, polishing, and UV curing [6].

### 1.3. AI Automation

Recent advances in AI [3] not only improve the overall design for 3D printing products and provide real-time monitoring and classification but also optimize the workflow with cloud services and cybersecurity enhancement. For the scope of this review, we focus on material- and process-related workflow optimization.

Goh, Sing, and Yeong reviewed the application, potential, and challenges of machine learning and AI for 3D printing previously [3]. However, we are not aware of any systematic attempt to summarize recent developments in AI for (3D) printing electronics. For any printing process, there is a vast parameter space involved, such as pre-printing treatment conditions, printing speed, printing temperature, printing environment, printing recipe, post-printing annealing, and packaging. Traditionally, these vast parameter spaces are explored manually or intuitively by a low-efficiency, trial-and-error process [3,7,8]. Electronics printing and 3D printing are even more complex, adding multiple electrical functionalities, multi-materials, and multi-dimensionalities to the process. AI algorithms can play a vital role in automating such processes efficiently.

Furthermore, the automation offered by electronics printing may be coupled with “digital twins” building, machine-learning-algorithm-based process optimization, and AI-driven discoveries to prepare us for the upcoming societal challenges on healthcare, global warming, energy production, and more [3].

### 1.4. Outline of This Review

In this review, we will first discuss printing electronics in general (e.g., contact and non-contact printing, electrostatic printing), then highlight recent progress in 3D printing electronics (including printing supercapacitors, sensors, batteries, and fuel cells). We will end with the current status of AI applications in 3D and electronics printing, summarizing popular algorithms and examples of optimization processes. It is important to point out that there are electronics printing methods based on lithographic and non-lithographic patterning. We will mainly be concerned with non-lithographic printing and patterning, meaning a direct ink or material is transferred, rather than those involving etching or photolithography. As far as we know, this current work represents the first systematic effort to summarize the recent trends in AI-assisted electronics printing, especially 3D electronics printing.

## 2. Printing Electronics

### 2.1. Research Trends

**Figure 1:**
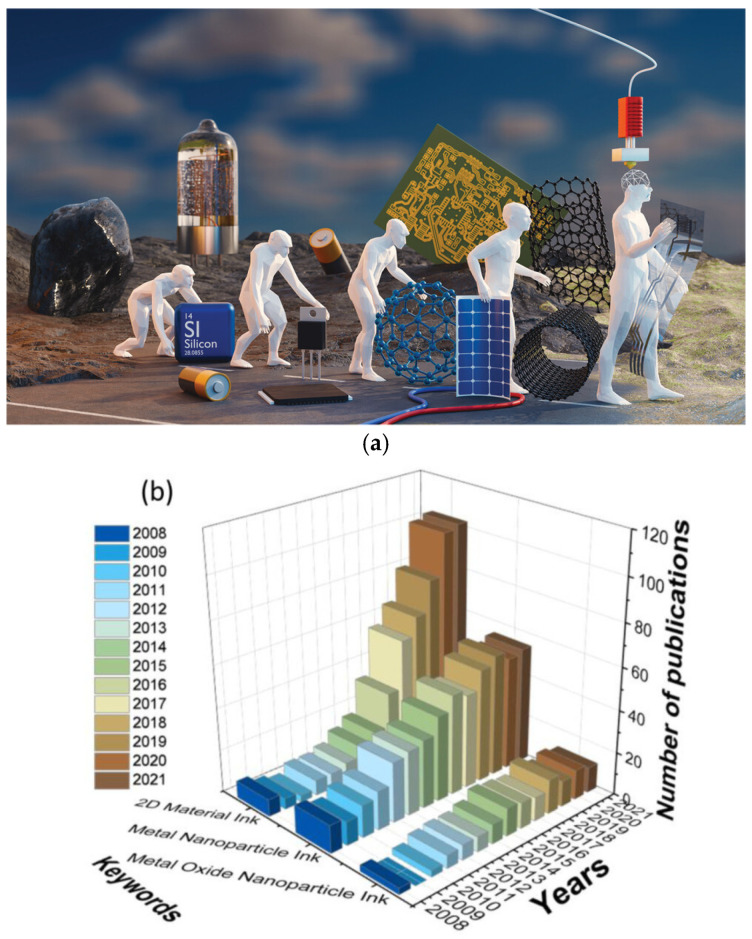
(**a**) Representation of the historical evolution of electronics: from the first use of Si-based materials to “printed electronics”. (Images and captions are used without changes from the reference [1], under Creative Commons BY-NC 4.0 License [9]). Bottom: Recent publications in the Web of Science database using keywords, (**b**) 2D material ink, metal nanoparticle ink, and metal oxide nanoparticle ink [4]. (Images and captions are used without changes from the reference [4], under Creative Commons 4.0 License [10]).

While silicon-based traditional electronics provide unmatched processing speeds for our computers and heavily rely on clean-room facilities such as lithography, printed electronics involve almost all components of conventional electronics while providing opportunities for low-cost personalization, flexibility, and better interface with biological and complicated surfaces [1]. Figure 1 shows an artistic rendering of electronics developments and the publication numbers in the Web of Science database related to printable electronics. A steady rising trend is clear over the years. Inks using 2D materials, metal, and oxide nanoparticles are compared in Figure 1, demonstrating the popularity of these inks [4].

### 2.2. Contact Printing

Contact printing is a cost-effective but versatile strategy for fabricating electronic devices, even on flexible and biodegradable substrates [11,12,13,14,15,16,17,18,19]. Typically, the process involves transferring an inked pattern or transferring material directly from the stamp to the substrate via pressure, sometimes with the aid of chemical or thermal activation [15,16]. Unlike photolithography, contact printing does not require sophisticated optics or high-energy radiation, making it suitable for roll-to-roll manufacturing and low-temperature processing environments essential for organic and flexible electronics. One prominent variant of contact printing is microcontact printing (µCP), a technique where an elastomeric stamp, usually made of polydimethylsiloxane (PDMS), is used to transfer self-assembled monolayers (SAMs) or functional inks onto a substrate [17].

Contact printing, based on the relative positioning of the printing area and non-printing area, can be divided into planographic (screen printing, offset printing), relief (letterpress, flexography), and intaglio (gravure printing) [20]. For example, in screen printing [21,22,23], the printing areas are flat with non-printing areas but pre-patterned with a mesh screen permeable to ink; in gravure printing, the printing areas are engraved below the flat surface of the non-printing areas; and in flexography, a flexible relief plate is adopted, with the non-printing areas below the printing areas [20].

Innovations like programmable contact printing using ballpoint pens loaded with silver nanoparticle (AgNP) and carbon nanotube (CNT) inks demonstrate the method’s flexibility, allowing sequential deposition of materials for disposable electrochemical sensors capable of detecting glucose [11]. In-tandem contact-transfer printing integrates contact and transfer methods to achieve high-quality electronic layers on flexible polyimide and biodegradable magnesium substrates, enabling transistors and UV photodetectors with superior performance [12]. In addition, debossed contact printing leverages paper’s porous microstructure: compressing the substrate creates relief structures that confine inks to raised regions, improving patterning resolution without hydrophobic coatings [13].

A contact printing system with independent control over normal and shear forces enables precise alignment of quasi-1D materials like nanowires (NWs), reducing batch-to-batch variability [14]. Automated sliding mechanisms ensure consistent NW transfer and alignment, which is essential for high-performance flexible electronics [14]. Contact printing is also integrated with electronics fabrication via nanotransfer printing (nTP) and nanoimprint lithography, targeting nanoscale or nanomaterial patterning at even higher resolution [18,19].

### 2.3. Non-Contact Printing: Main Techniques

Non-contact printing revolutionized the fabrication of stretchable electronics by enabling precise, damage-free deposition of optoelectronic or functional materials on a variety of substrates [24,25,26]. Non-contact printing methods for electronics represent a class of additive manufacturing techniques where the material is deposited onto a substrate without any physical contact between the printing tool and the surface, often with digital programming that enables maskless patterning for reduced cost and turnaround time for prototyping and customizable manufacturing.

**Figure 2:**
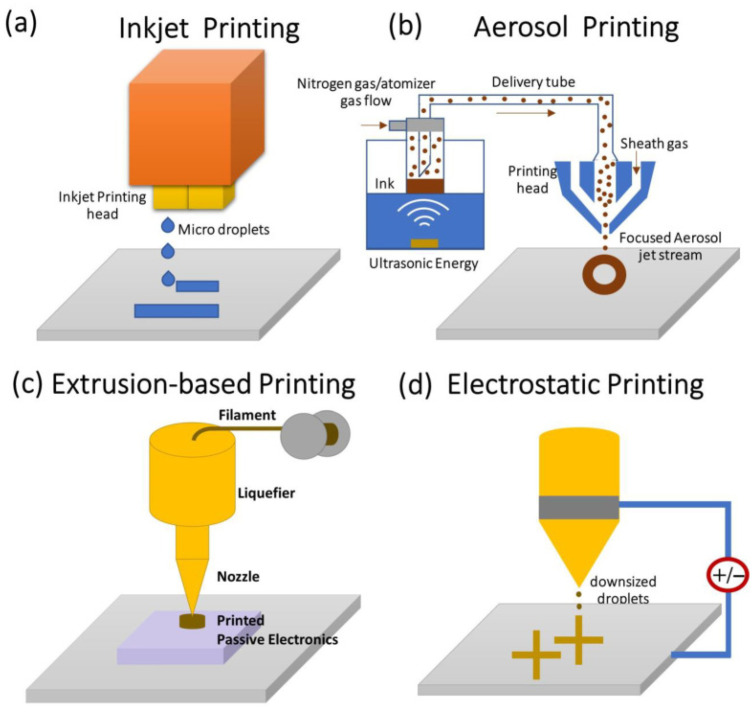
Printing technologies using conducting ink (**a**) inkjet printing; (**b**) aerosol printing; (**c**) extrusion-based printing; and (**d**) electrohydrodynamic printing [4]. (Images and captions are used without changes from the reference [4], under Creative Commons 4.0 License [10]).

Non-contact printing techniques (Figure 2) include inkjet, aerosol, filamentary printing, or electrostatic printing, sometimes with UV-light curing [4,27,28,29]. A typical nozzle size for Inkjet printing is 20 microns, an order of magnitude smaller than that of aerosol-jet printing (100–300 microns). In contrast, the nozzle sizes for electrostatic printing may range from 0.2 to 100 microns [4]. Inkjet printing ejects non-viscous droplets that collapse or evaporate on substrates, electrostatic printing drives with surface-tension-limited meniscus formation under an applied electrical field, and aerosol printing relies on a carrier gas to bring atomized ink from reservoir to print head, sheath gas to drive the aerosol to substrates, and the focusing ratio between sheath and carrier gas flow rate to control printing resolution (Figure 2a,b,d) [4]. Extrusion printing (Figure 2c) can be used for 2D and 3D object manufacturing, which will be detailed in the next section.

Inkjet printing (IJP) with drop-on-demand mechanisms was used to pattern conductive silver inks on polyethylene terephthalate (PET) and glass, achieving surface conductances up to 2.78 Ω^−1^/cm^2^ [26]. Aerosol jet printing (AJP) extends capabilities to inks of higher viscosity (up to 1000 cP, from 5–20 cP for IJP) and sub-10 µm line widths, critical for interconnects in wearable sensors and radio-frequency identification tags [24]. Both methods excel in multilayer printing, with IJP demonstrating linear thickness scaling and AJP enabling conformal coatings on textured surfaces.

### 2.4. Non-Contact Printing: Electrostatic Printing and Electrospinning

Electrostatic printing and electrospinning have been an important area for next-generation energy and medicine applications [30,31,32,33,34,35]. Both electrostatic printing (Figure 2d) and electrospinning are techniques of utilizing electric fields to manipulate formulated precursors, thus extruding materials. Although electrospinning generates nano- to submicron-scale fibers in a membrane form with high intrinsic porosity and surface area, electrostatic printing allows the deposition of materials in a precise and controlled manner [34,35]. The shared apparatus in these two techniques is the charged nozzle and collector bearing the opposite charge. The charged nozzle facilitates the charges of chemical components in the formulation, critical to the transformation from droplets to jets (Taylor cone), the trajectory of formulated materials to the target, and the subsequent adhesion to the substrates [34,35,36]. In electrospinning, the electric field changes lead to various fiber morphologies with tunable parameters in the membrane structure and function [36]. In electrostatic printing, in addition to the above-mentioned effect, it also plays a critical role in printing resolution due to its impact on droplet size and the direct writing nature of this technique [34].

Moreover, with the early investigation of electrospinning in the 1900s and recent developments in modern electrospinning techniques [37], commercial bench scale, pilot scale, and manufacturing-grade electrospinning apparatus with controlled environments became available for both R&D work and production. In contrast, the electrostatic printer is still in its infancy, offering advantages in electronics fabrication with more precise and controlled patterns [34].

Wang et al. reviewed the recent progress of electrospun nanofiber based soft electronics, and these fibrous membranes exhibit broad applications in sensors, photodetectors, energy storage, and transistors with known primary advantages of large surface area and flexibility. There are also limitations in fiber formation capabilities among common chemicals used in electronics, and there are challenges with blends and composites [35]. Some examples of possible applications of the resultant nanofiber assemblies in energy storage include: (1) Tubular carbon network for enhanced battery electrodes via electrospun polymer templates, (2) fillers to enhance ion conductivity of solid electrolytes, (3) porous, flexible membranes for high-performance separators and stand-alone electrodes, and (4) 3D host for lithium metal and sulfur [30].

### 2.5. Opportunities and Challenges

A few previous reviews summarized recent developments in printed electronics [1,20,31]. These include but are not limited to, printing conductive structures (Ag, Au, Cu, Al, Ni, carbon nanotubes), semiconductor layers (Poly 3-hexylthiophene, functionalized pentacenes, CuPc, ZnO, TiO_2_, Cu_2_O, SnO_2_, In_2_O_3_, MoS_2_, quantum dots), dielectric materials (Poly 4-vinylphenol or PVP, Polymethyl methacrylate or PMMA, BaTiO_3_), on flexible insulating substrates (Polyethylene terephthalate or PET, Polyimide, Poly (ethylene naphthalate or PEN).

Compared to conventional processes, electronics printing can be more flexible and cost-effective. At the same time, the sizes of printed devices, such as transistors, can be much larger than their lithography-made counterparts [1]. In addition, the conductive inks need further improvement to decrease power consumption, reduce dependence on expensive components such as silver, and resolve issues caused by cracking, diffusion, and oxidation [1].

Traditional battery manufacturing involves (1) slurries preparation, (2) tape casting on foils of current collectors, (3) calendaring, (4) electrode cutting, (5) stacking and packaging, and (6) electrolyte injection, with limited opportunities for miniaturization [2]. On the other hand, additive manufacturing is capable of fabricating complex structures with low waste and high energy densities. The corresponding challenges include achieving a balance between rheological, mechanical, and electrochemistry behavior, the possible need to prepare filament or ink in-house, dealing with air- or moisture-sensitive components during printing, etc. [2].

## 3. Three-Dimensional Printing of Electronics

### 3.1. Developments in 3D Printed Electronics

Additive manufacturing (AM) can build objects one layer at a time using techniques such as jetting, lamination, extrusion, powder fusion, deposition, and photopolymerization [38,39,40,41,42]. Compared to traditional subtractive manufacturing, AM is much less reliant on supply chains and tooling while excels in complicated geometries and customization [38,42]. The various electronics that can be manufactured via additive manufacturing include transistors, diodes, multilayer circuits, solar cells, interconnects, antennas, batteries, supercapacitors, fuel cells, and sensors [43,44,45,46,47,48,49].

**Figure 3:**
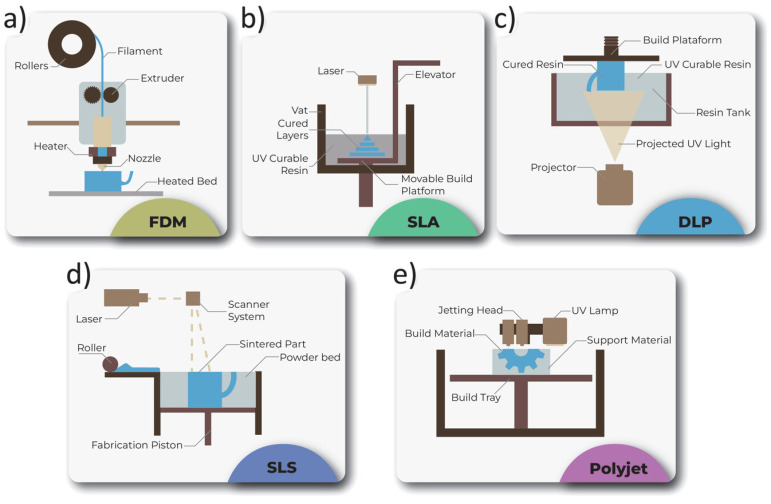
Illustrated scheme of: (**a**) FDM; (**b**) SLA; (**c**) DLP; (**d**) SLS; and (**e**) Polyjet. (Figures and Captions above are from reference [1] without change, under Creative Commons BY-NC 4.0 License [9]). These are popular 3D printing techniques: FDM stands for fused deposition modeling, SLA for stereolithography, DLP for digital light projection, SLS for selective laser sintering, and Polyjet for polymer jetting.

A number of previous reviews already summarized recent developments in 3D printed electronics [3,4,40,41,44,45,46,47,48,49]. These included 3D printing energy harvest and storage devices, antennas, electronic components (such as transistors and capacitors), biosensors, wearable electronics, and soft robotics [1,3,4,40,41,44,45,46,47,48,49]. Figure 3 illustrates popular 3D printing techniques: FDM stands for fused deposition modeling, SLA for stereolithography, DLP for digital light projection, SLS for selective laser sintering, and Polyjet for polymer jetting [1]. Although 2D electronics printing prevails at reducing waste and high resolution, 3D electronics printing provides unprecedented whole-structure-printing with a multitude of possibilities in directed deposition, binder/material jetting, extrusion, powder bed fusion, photopolymerization, and lamination [1]. Table 1 provides a quantitative comparison between different 3D printing technologies in aspects such as accuracy (resolution), compatibility, surface, speed, strength, limitations, and cost.

FDM, based on extrusion and designed for plastics, eutectic metals, and ceramics, uses a fabrication speed of about 0.05 cm per hour, a typical resolution of 50 elements per millimeter, providing easy, low-cost, multi-material, but low-speed and low-resolution printings [1]. SLA, based on photopolymerization and designed for photo-cross-linkable polymers, uses a typical fabrication speed of about 1.5 cm per hour, a typical resolution of 3000 elements per millimeter, providing high-resolution but high-cost printings [1]. DLP, also based on photopolymerization and designed for photopolymers, uses a typical fabrication speed of 2 cm per hour, a resolution of about 3000 elements per millimeter, providing fast, high-resolution, but relatively expensive printings [1]. SLS, based on sintering and designed for powdered plastics, metals, and ceramics, uses a fabrication speed of about 2.5 cm per hour, a typical resolution of 200 elements per millimeter, providing high-strength and high-resolution, but high-cost, possibly porous printing [1]. Polyjet, based on inkjet and designed for liquid photopolymers, uses a typical fabrication speed of about 0.4 cm per hour, a resolution of about 15,000 elements per millimeter, providing high-resolution but expensive, low-strength printing [1].

For FDM, which relies on the extrusion of filaments, the print quality can be affected by filament properties, layer height, shell thickness, nozzle and substrate temperature, print speed, infill, and pattern structure [1,3,4,40,41,44,45,46,47,48,49]. For 2D direct ink writing (DIW), a meniscus at the nozzle tip is pushed into formation with pneumatic pressure. With sufficient substrate wetting, shear drives printing in a 2D plane [46]. The pressure inside the ink nozzle, the nozzle-to-substrate distance, and the substrate wettability are among the most important printing parameters. For 3D DIW, the ink may be expected to maintain structure on the substrate [46]. Viscosities as a function of post-printing solidification, sintering, or annealing are also important, along with substrate temperature and writing speed.

The 3D photocuring methods involve exposing the liquid resin to a specific photon wavelength, yielding high-resolution structures [46]. Photon wavelengths, the corresponding penetration depth, curing speed, and substrate movement can affect the overall printing process. The photopolymer viscosity, stability, and mechanical properties are all critical to photo-curing-based 3D printing [46].

### 3.2. Supercapacitors by 3D Printing

Traditional manufacturing techniques often limit the design freedom of supercapacitor electrodes, whereas 3D printing enables precise spatial control over material deposition, thereby improving surface area utilization and energy density [57,58]. Among the various 3D printing techniques, direct ink writing (DIW) is particularly well-suited for supercapacitor fabrication because of its ability to include conductive fillers like graphene, carbon nanotubes (CNTs), or MXenes in a printable ink [57,58]. Interdigitated electrode architectures, which can be readily fabricated via 3D printing, offer a compact layout that shortens ion diffusion paths and improves the power density of supercapacitors.

**Figure 4:**
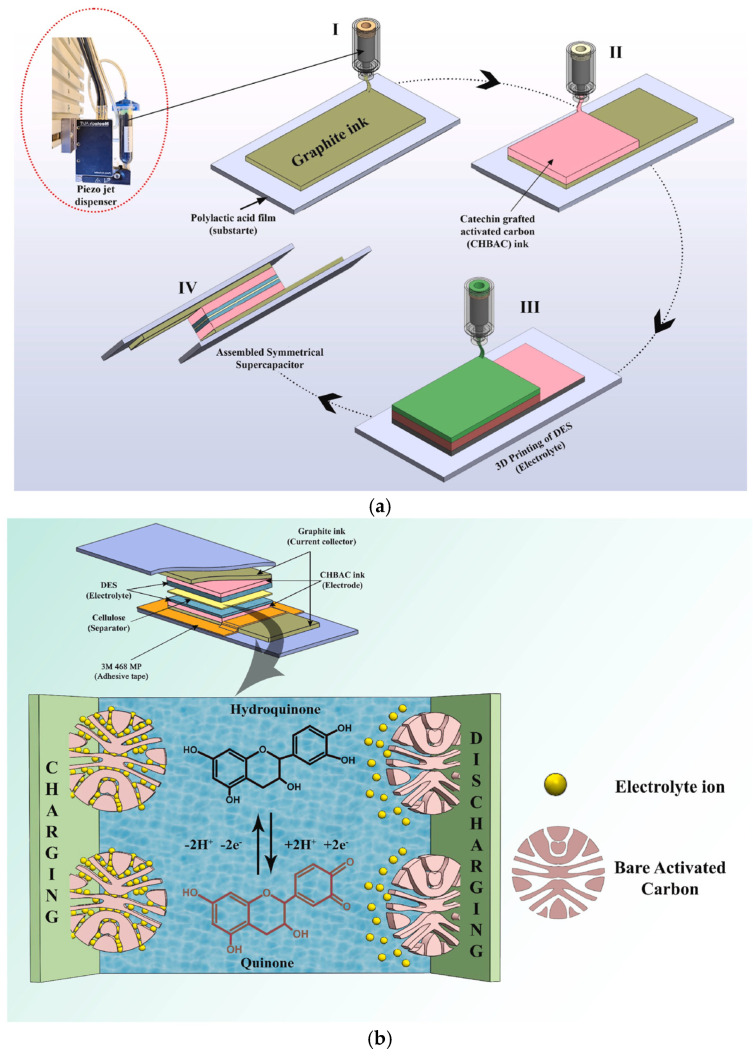
(**a**) Schematic illustration depicting the steps entailed in the fabrication of the SC. (**b**) Charge storage mechanism in CHBAC electrode. (CHBAC refers to catechin-grafted activated carbon; SC for supercapacitor; images and captions are used without changes from reference [61], under Creative Commons 4.0 License [10]).

Flexible, safe, light, stable supercapacitors with excellent power density and cyclability can be achieved via contact or non-contact printing of customer-designed current collectors, substrates, electrodes, as well as electrolyte materials [59]. Choi et al. demonstrated a solid-state, paper-based, flexible supercapacitor by printing carbon nanotubes, ionic liquid, and photo-crosslinkable triacrylate polymer electrolyte, along with a cellulose nanofibril-based primer layer and Ag-nanowires-based electrodes [60]. Mevada et al. enhanced activated carbon with catechin hydrate grafting and printed choline chloride/urea deep eutectic system (DES)-based electrolytes to a capacitance of 75 F/g, potential window of 2.0 V, 90% cycling stability after 10k cycles (Figure 4) [61]. To tackle high capacitance, large mass loading, structural complexity, and a high degree of system integration, 3D-printing techniques need continued adaptations to achieve highly porous structures in interdigital, modular, or integrated circuit designs for their potential in various supercapacitor usage scenarios [62].

Key challenges exist in 3D printing supercapacitors. Those include achieving high mass loading while preserving print resolution, ensuring cycling stability, and integrating printed supercapacitors into wearable systems [57]. Emerging strategies to address these challenges include the development of stretchable inks, hybrid printing processes combining DIW with inkjet or aerosol jet printing, and in situ post-treatments such as laser sintering and thermal annealing to enhance conductivity and adhesion [58]. As material formulations and printer technologies evolve, 3D-printed supercapacitors are poised to play an important role in next-generation portable and structural energy storage devices.

### 3.3. Sensors by 3D Printing

Three-dimensional printing for sensor fabrication has emerged as a transformative approach in the development of next-generation sensing devices [63,64,65,66,67,68,69,70,71]. A layer-by-layer additive manufacturing process enables the creation of multifunctional components and customizable designs [65,66]. Unlike conventional sensor fabrication methods, which often involve multiple stages in cleanroom facilities, 3D printing offers a low-cost, scalable, and decentralized platform for sensor prototyping and production. Direct ink writing and inkjet printing are of intense interest for printing functional, nanomaterial-based inks (such as silver nanoparticles, carbon nanotubes, and graphene) that form the active elements of resistive, capacitive, piezoelectric, or electrochemical sensors [63,64,65,66,67,68,69,70,71]. Multi-material and hybrid 3D printing enable the integration of structural and functional materials, allowing for miniaturized co-fabrication of various sensor components.

**Figure 5:**
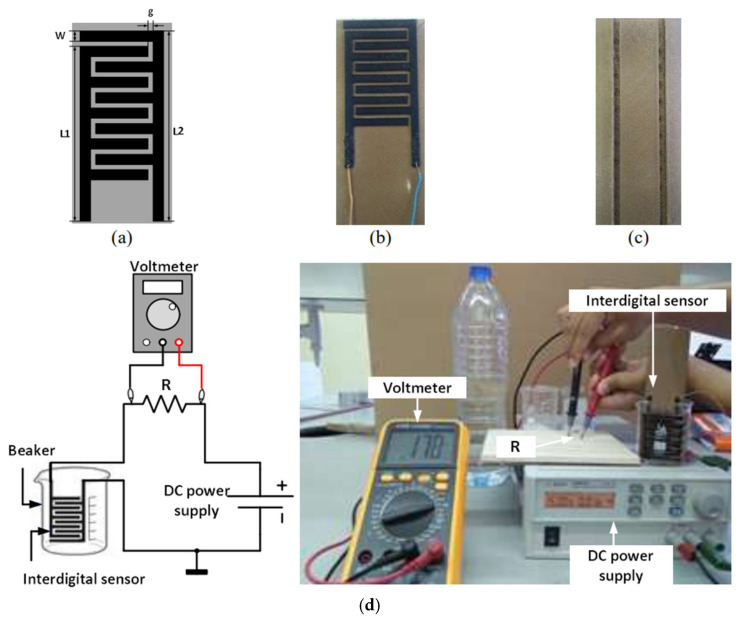
Interdigital conductive ABS sensor (**a**) structural layout (**b**) front view of fabricated sensor (**c**) back view of fabricated sensor (W = 4 mm, L1 = 65 mm, L2 = 70 mm and g = 2 mm). (**d**) Salt and sucrose concentration sensing. (Images and captions are used without changes from reference [74], under Creative Commons [75]).

Compared to silicon or inorganic semiconductor-based rigid sensors widely used in current communication systems (such as cell phones) and robotics, flexible sensors are necessary in future healthcare and internet-of-things scenarios [72]. Wang et al. reviewed processes and applications of low-cost inkjet-printable sensors, which can be light, flexible, and fabricated with mass production [73]. Ponan and Harnsoongnoen demonstrated that 3D printing with conductive polymer filaments can make an interdigital sensor for salt and sucrose on an insulating substrate (Figure 5) [74]. The sensing was based on electrical responses using a direct current.

Challenges in such technologies remain in terms of material compatibility, resolution, and long-term reliability of 3D-printed sensors [63,65,66,69,70]. Achieving uniform dispersion of nanomaterials within printable matrices, maintaining structural integrity under mechanical stress, and ensuring repeatable sensor performance over time are some critical areas of ongoing research efforts [69,70,71]. Emerging trends include 4D printing (time-responsive sensors), self-healing sensor structures, and biodegradable sensors for transient electronics [63,64,65,66,67,68,69,70,71]. As materials science and printing technologies converge, 3D printing is poised to revolutionize how sensors are designed, manufactured, and deployed across diverse sectors.

### 3.4. Batteries by 3D Printing

Battery printing can enable safe, environmentally friendly, flexible forms of energy storage [76,77,78,79,80,81]. While traditional battery manufacturing relies on planar electrode designs, 3D printing allows for interdigitated and porous structures to increase electrode–electrolyte interface and shorten paths of ion diffusion, with enhanced volumetric energy and power densities [76,80,82,83,84,85]. Additive manufacturing of batteries is suitable for miniaturized electronics, implantable devices, remote sensors, transmitters, smart cards, and the Internet of Things (IoT) networks, with design freedom, higher (area or volume) energy densities, larger power density, and reduced cost, via techniques such as direct ink writing (DIW), fused deposition modeling (FDM), inkjet printing (IJP), and stereolithography (SLA) [2].

Direct ink writing has been an extensively explored printing method for battery electrodes because it can utilize high-viscosity inks and electrochemically active materials [76,80,82,83,84,85]. Using DIW, Wei et al. demonstrated one of the earliest 3D-printed lithium-ion microbatteries of interdigitated anode and cathode structures [86]. The batteries exhibited good areal capacities and rate performance, highlighting the advantages of 3D architectures for microelectronic devices. To ensure printability with DIW, the inks must maintain structural integrity upon deposition and exhibit proper rheological behavior during printing [76,80,82,83]. For example, Martinez et al. [87], developed a multi-process to 3D print sodium-ion batteries, in which DIW is used for positive electrodes and vat photopolymerization (VPP) is used for gel polymer electrolytes. With Na_0.44_MnO_2_ electrodes, 3D printed electrolytes show room-temperature ionic conductivities up to 3.3 × 10^−3^ S·cm^−1^ and a stability window of 4.8 V [87]. In addition, Ma et al. used DIW-based Printing to fabricate planar, flexible sodium-ion microbatteries with microelectrodes of up to 1200 µm thickness, 4.5 mAh cm^−2^ areal capacity, 3.6 mAh cm^−2^ rate capability, for as long as 6000 cycles [88].

Issues such as achieving high energy density, maintaining mechanical integrity during cycling, optimizing ion and electron transport pathways, and ensuring compatibility between printed layers are key obstacles [80,85,86]. Continued innovation in materials chemistry, rheological control, and multi-material printing is critical for realizing 3D-printed, high-performance batteries for applications ranging from IoT devices to flexible electronics.

### 3.5. Fuel Cells by 3D Printing

Furthermore, 3D -printing-enabled fuel cell technologies are gaining traction. As a clean energy carrier, hydrogen is undergoing an unparalleled surge in interest, with numerous countries launching national hydrogen strategies that are accelerating the emergence of a global hydrogen economy [89]. Target applications range from ammonia production and steel refining to synthetic fuels from captured CO_2_ or electricity through a fuel cell [90,91,92]. Fuel cells are being extensively developed for heavy-duty transportation, such as Class 8 trucks, trains, ferries, and airplanes [93].

Three-dimensional printing has emerged as a transformative technology in the development and production of fuel cells [94,95]. This approach enables the creation of complex and customized components that enhance the performance, efficiency, and cost-effectiveness of fuel cell systems for the following reasons: (1) One of the primary advantages of 3D printing in fuel cell applications is the ability to produce intricate parts, such as proton exchange membranes (PEM), flow field plates, and gas diffusion layers, with high precision. Traditional manufacturing methods often struggle with the complexity of fuel cell components, but 3D printing can generate complex geometries that optimize the flow of reactant gases and improve overall fuel cell efficiency. (2) Three-dimensional-printed flow field plates are a key area of interest, as they can be designed with optimized channel structures to ensure even distribution of reactants, reducing energy losses and enhancing the fuel cell’s performance. (3) 3D printing enables the design of lightweight and compact parts, essential for fuel cell applications in sectors like automotive and aerospace, where space and weight constraints are critical. (4) The rapid prototyping capabilities of 3D printing also accelerate the development process, allowing for faster iteration and testing of new designs, significantly shortening the development cycle. (5) Additionally, the ability to use various materials in 3D printing, such as metals, ceramics, and polymers, offers the potential for producing highly durable and corrosion-resistant fuel cell components. This versatility also aids in optimizing the performance and lifespan of fuel cells under varying operating conditions.

Despite the numerous benefits, the following challenges remain: (1) material limitations related to the printing process, (2) scalability, and (3) the need for post-processing 3D-printed parts. Nevertheless, ongoing advancements in 3D printing techniques and materials are expected to overcome these barriers. Lira et al. reported a solid oxide cell (SOC) benefited from advanced ceramic 3D printing methods such as stereolithography (SLA) and robocasting,[96] which allow for the creation of complex shapes, improved interfaces, and reduced material waste. This study focuses on large-area SOCs (25 cm^2^) fabricated using SLA 3D printing, with electrolyte supports made of yttria-stabilized zirconia (8YSZ) and composite electrodes of nickel (fuel electrode) and lanthanum strontium manganite (oxygen electrode). The printed electrolytes featured advanced honeycomb geometries for enhanced performance. In fuel cell mode, the cells generated up to 3.5 W with a peak power density of 240–220 mW/cm^2^ at 950 °C, and in the electrolysis mode, they provided 7.3 W with a current density of 340 mA/cm^2^ at 1.3 V. A long-term degradation test at 900 °C over 1150 h showed minimal degradation of 11 mV/kWh.

In comparison, solid oxide fuel cells have also been manufactured using traditional contact and non-contact printing methods. Han et al. developed an inkjet-printed submicron-thickness ceramic electrolyte that maintained a stable microstructure during electrochemical testing [97]. Somalu et al. reviewed the application of screen printing in fabricating solid oxide fuel cell electrodes and electrolytes with thicknesses between 10 and 100 µm [98]. The oxide particles, binder, dispersant, or solvent in the screen-printing ink recipes contribute to their overall rheology, as well as mechanical, electrical, and electrochemical performances.

## 4. AI in 3D and Electronics Printing

### 4.1. The Rise of AI

The automation of 3D printing and electronics printing can be driven by building high-quality databases and “digital twins”, as well as AI-powered discoveries. Here, we present the current status of AI applications in 3D and electronics printing, summarizing popular algorithms and examples of optimization processes.

To enhance printing quality and efficiency at another level [3,7,8,99,100,101,102,103], machine learning algorithms can be used in planning, design automation, property prediction, process monitoring and optimization, quality control, maintenance scheduling, and defect detection [104,105,106,107,108,109]. In situ or customized additive manufacturing, with bio-electroactive inks and open-source AI algorithms, can lead to wearable and smart biosensors at a fraction of the current cost for a better healthcare future via efficient diagnostics and regenerative medicines. Previous reviews summarized recent developments of AI for 3D printing [3], and 3D printed electronics [40]. Here, we combined available progress to provide an overview of AI-assisted 3D printing and printing electronics (including 3D electronics printing).

### 4.2. AI Applications

**Figure 6:**
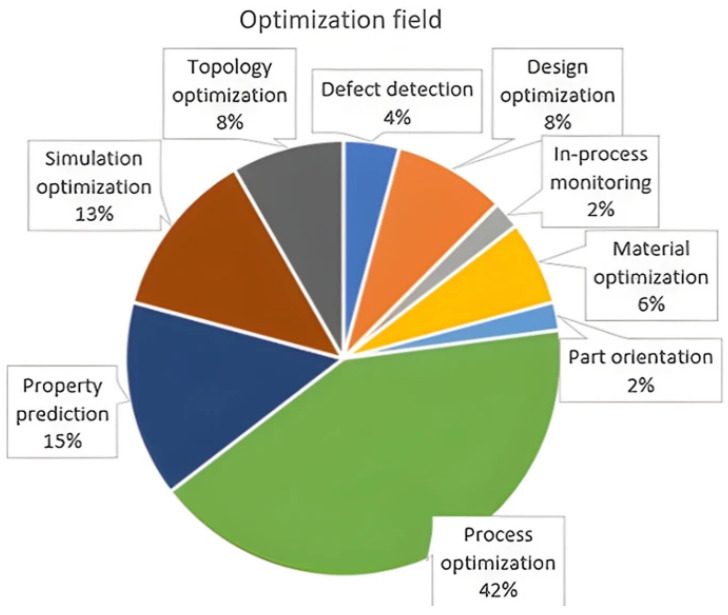
Field of application of optimization in the 48 publications selected from the relevant literature [110] (The image and caption are used without changes from the reference [110], under Creative Commons 4.0 License [10]).

Figure 5 analyzes the application areas of AI models that enhance 3D printing. For fused diffusion modeling (FDM), nozzle temperature, flow rate, and pressure can be significant as model inputs or features, while parameters such as laser power and scan speed are critical for Powder Bed Fusion (PBF). Consequently, 42% of the AI optimizations in Figure 6 deal with the manufacturing process.

Other essential subjects of AI algorithms include (1) quality predictions of the printed products (for example, roughness and mechanical strength), (2) simulation optimization for reduced computational costs, (3) design, and (4) topology optimization.

The integration of artificial intelligence (AI) in electronics printing has enabled new levels of precision, adaptability, and process optimization, particularly in inkjet, screen, or 3D printing of electronic materials. The following aspects showcase the recent progress of AI in printing electronics.

(1) Inkjet path optimization

A prominent example of AI integration is in inkjet path optimization, where deep reinforcement learning (DRL) has been employed to reduce inefficiencies in nozzle travel and droplet deposition. Kim et al. demonstrated that, from the rheological properties of the ink and parameters of the waveform, DRL can dynamically forecast the inkjet drop velocity and jetting morphology for quantum dot inks, with a target drop velocity of 3 ms^−1^ achieved within 20 steps [111]. Xiong et al. proposed a multi-algorithm planning system to pattern organic light-emitting diode arrays [112]. Three algorithms are used for three inkjet planning needs: a graph convolutional neural network for high overall performance, a greedy algorithm for high solution quality, and an interval search algorithm for short solution time. Additionally, Proximal Policy Optimization was used to build an algorithm selection network to validate the multi-algorithm planning system with an in-house NEJ-PRG4.5 inkjet machine [112].

(2) Defect prediction

Another critical application of AI lies in defect detection and prediction using computer vision and deep learning models. High-speed electronics printing can produce defects such as missing lines, satellite droplets, and ink bleeding, which are difficult to detect using traditional threshold-based inspection systems [113,114,115].

Polomoshnov and coworkers [113] proposed an automated image-based approach to analyze inkjet-printed conductive traces. A dataset of over 5000 was built, with images of conductive structures and corresponding physical characteristics. A convolutional neural network (CNN) is used to predict physical characteristics from acquired images. It is found that an overly sophisticated CNN does not improve accuracy. A validation accuracy of 0.883 was achieved after the 4th epoch and 0.880 after the 24th epoch, with a training time of 2.25 h per epoch [113]. Liu et al. [115] proposed a model based on YOLOv5 to efficiently detect printing defects. The C3 module was replaced with a C3-Deformable Convolution Network (DCN) module, enhancing the detection of narrow and elongated defects. Large Selective Kernel and RepConv modules were used in the feature fusion network, with a Normalized Gaussian Wasserstein Distance and Efficient IoU (Intersection over Union) based loss function to detect small targets. Model pruning was used to reduce parameter size for high-speed detection. The final model achieved an enhancement of up to 2.7% in mean average precision at a threshold IoU value of 0.5 (mAP@0.5) and a 20% inference time reduction, as compared to the YOLOv5 baseline [115].

Gafurov et al. [114] integrated 19 U-Net-based models with roll-to-roll screen printing to detect smearing, a main defect of screen printing. The model parameters were between 8000 and 30 million, with validation mean IoU up to 95.1%.

(3) Parameter reverse inference

AI has also been proven effective in parameter reverse inference, a process in which desired printing outputs (e.g., line width, resistivity, or layer thickness) are used to infer optimal process settings [116]. Roach et al. developed a deep invertible neural network (DINN) that solves both forward optimization and inverse inference problems with in-situ computer vision (CV) [116]. In their study, a direct ink write (DIW) system with motorized stages moves a base plate in the X-Y zone. A syringe pump moves in the *Z*-axis to print a silicone elastomer onto a Teflon-coated plate. In the DINNs, the input and output are in the same sizes as the hidden layers, using leaky ReLU activations. The inputs (or printing parameters) are nozzle diameter D, printing speed V, extrusion rate A, and the distance from the substrate H. The output is the printed and measurable line width. Images of these printed lines were taken using a digital computer-vision-powered camera system at a 45-degree viewing angle to the nozzle. A total of 196 lines were printed with a range of DIW printing parameters with corresponding real-time width measurements to train DINN models with 1–9 hidden layers. At a learning rate of 5 × 10^−4^, these DINNs converged in 10k to 100k epochs for 30–50 µm line widths. The trained reversible DINN was then asked to predict printing parameters for a 300 µm line. The DINN predicted a D of 1.19 mm, H of 1.23 mm, V of 75 mm/s, as well as a parameter A of 0.34 mm/s. With autonomous optimization, a pre-set line width of 300 µm was printed within 2 s. Printing defects were then automatically corrected after CV identification without user intervention in real time [116].

### 4.3. Champion Models

**Figure 7:**
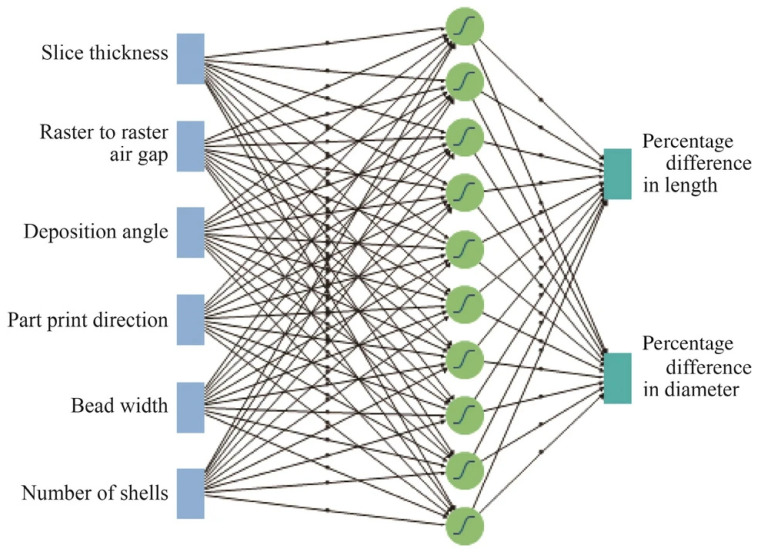
Schematic example of the ANN [117] (The image and caption are used without changes from the reference [117], under Creative Commons 4.0 License [10]).

An artificial neural network (ANN) was used to simulate melting kinematics and filament flows in FDM processes [117]. Mohamed et al. used FDM settings as inputs to predict the resultant print dimensions [117]. The ANN model in Figure 7 showcases the internal construct of the algorithm. The ANN algorithm uses features such as slice thickness, raster-to-raster air gap, deposition angle, printing direction, bead width, and shell number to predict the length and diameter changes of the 3D-printed cylinders.

**Figure 8:**
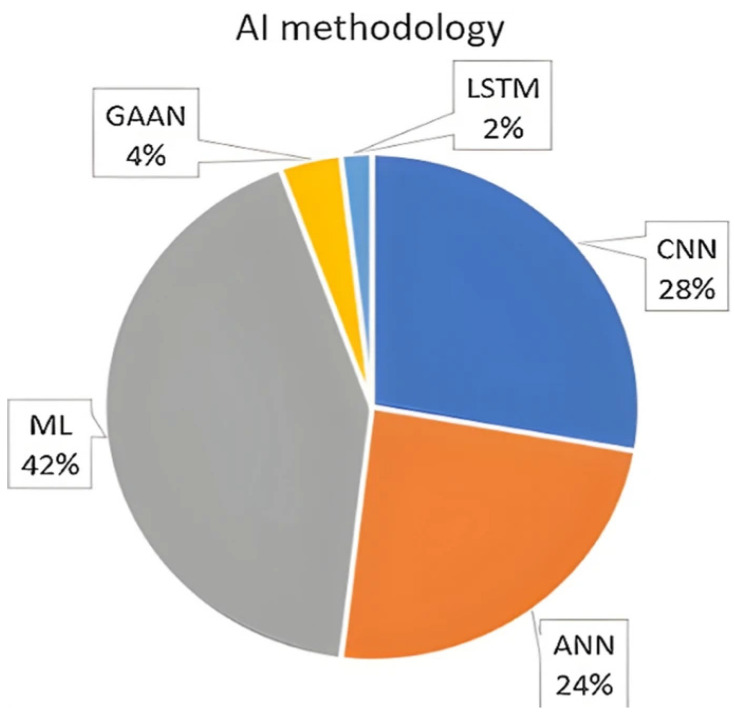
AI methodologies applied in the 48 publications selected from the relevant literature [110] (The image is used without changes from the reference [110], under Creative Commons 4.0 License [10]).

Some of the popular algorithms used in AI-aided manufacturing include support vector machine (SVM), Bayesian networks, and deep learning (including computer vision and natural language processing) [118]. Figure 8 has a pie chart of AI algorithms for the above-mentioned (Figure 5) publications on 3D printing optimizations [110]. In this chart, deep learning (DL) models are specified separately, but other regression and classification models are under the general machine learning (ML) group. DL models in the chart include artificial neural network (ANN), convolutional neural network (CNN), Generative Adversarial Artificial Networks (GAAN), and Long Short-Term Memory (LSTM) networks [110]. GAAN relies on a generator and discriminator to achieve high-performance unsupervised learning, while neural networks are designed for supervised learning [118]. Recurrent neural networks, such as LSTM and Attention models, provide the capability to selectively discard or keep information from previous frames [118]. Table 2 compares different AI models in 3D printing applications with regard to accuracy, interpretability, training need, use cases, adaptability, and computational cost.

Different AI algorithms have distinctively different strengths and application scenarios in 3D printing [119,120,121,122,123,124,125,126,127,128,129]. SVM models excel in low-data scenarios and offer strong generalization, though they lack advanced feature learning capabilities [127]. ANNs can model complex non-linear relationships in the process-to-property mappings but require larger datasets and tuning [129]. CNNs are dominant in image-based quality control and in-situ monitoring [119]. GANs, although resource-intensive, are powerful in generating synthetic data for augmenting datasets and printable geometry designs [122].

The ability to adapt to new material recipes or printing setups can vary greatly for the champion models in 3D printing [119,120,121,122,123,124,125,126,127,128,129]. For SVM, adapting to new uses requires careful selection of kernel functions and feature engineering [127]. It can be robust for moderate scenario shifts but may not be easily adaptable for radically new materials or geometries. For ANN, adaptability is limited by the need for large retraining datasets and possible architecture redesign for new applications [129]. Bayesian Optimization can be highly adaptable to new materials or printers, as it can efficiently explore new parameter spaces with minimal prior data [127]. Pareto Methods are adaptable to new trade-off objectives but constrained by how objectives are defined [120]. Random Forest can be highly adaptable to new data types and applications, robust against overfitting, and can mix categorical and continuous features [119]. GAN, however, has Limited adaptability due to the need for large, domain-specific datasets for retraining, but it excels within a single domain [122].

**Figure 9:**
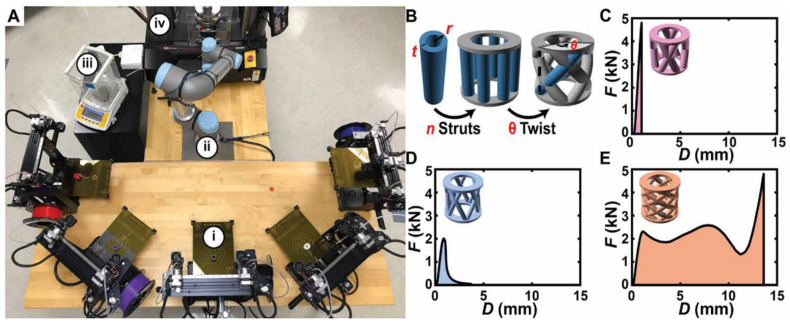
BEAR for studying the mechanics of additively manufactured components. (**A**) Experimental system composed of (**i**) five dual extruder fused deposition modeling (FDM) printers (M3, MakerGear), (**ii**) a six-axis robotic arm (UR5e, Universal Robotics), (**iii**) a scale (CP225D, Sartorius), and (**iv**) a universal testing machine (5965, Instron Inc.). (Photo credit: Aldair E. Gongora and Bowen Xu, Boston University). (**B**) Model “crossed barrel” family of parametric structures with two circular platforms that are held apart by a series of n hollow columns of outer radius r and thickness t and that are twisted with an angle θ. Force F and corresponding displacement D from the testing of (**C**) a crossed barrel that did not yield before ~5 kN (designated too strong), (**D**) a crossed barrel that failed in a brittle manner (designated “brittle”), and (**E**) a crossed barrel that exhibited appreciable strength after an initial yield point (designated “ductile”). (Figure and Caption from reference [130] without change, under Creative Commons Attribution 4.0 International License [10]).

**Figure 10:**
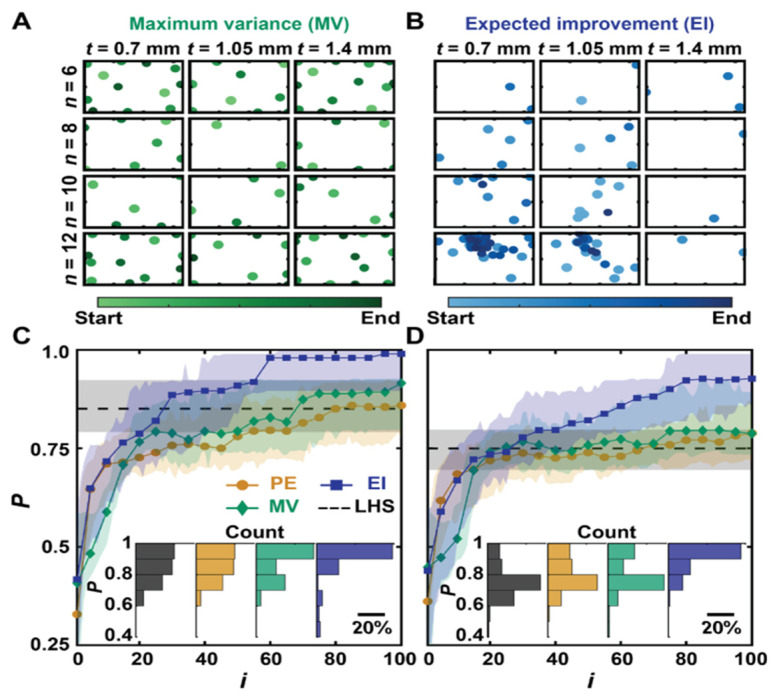
Simulated learning using BO. Distribution of experimental points when guided using (**A**) MV and (**B**) EI decision-making policies. The color gradient indicates the start and end of the campaign. Performance P versus experiment number i of simulated Bayesian campaigns with noise added to each simulated measurement drawn from a zero-mean Gaussian with (**C**) SD σ = 0.1 J and (**D**) σ = 5 J. EI- and MV-guided campaigns are benchmarked against PE, and the average result of selecting 100 experiments using Latin hypercube sampling (LHS). Shaded regions correspond to the middle two quartiles of 100 simulated campaigns. The inset bar charts show the distribution in P at i = 100. (Figure and Caption from reference [130] without change, under Creative Commons Attribution 4.0 International License [10]).

In AI-automated 3D printing, Gongora et al. [130] developed a Bayesian experimental autonomous researcher (BEAR) to combine high-throughput automated experimentation (Figure 9) and Bayesian optimization (Figure 10). This achieved a 60-fold experimental iteration reduction in an effort to maximize the toughness of the printed structure, as compared to a grid search. It is noted that this was achieved with limited experimental data availability. An automated polymer analysis and discovery array (PANDA) combined high-throughput fluid handling, electrodeposition, electrochemistry, and optical measurements in 96-well plates, with a goal of maximizing electrochromic switching of conductive polymer with Bayesian-optimization generated parameters (e.g., monomer concentration, deposition time and voltage) [131]. We expect that the future adaptation of similar approaches will benefit electronics printing significantly.

### 4.4. Optimizing Property and Process Simultaneously

It is important to point out that for both additive and subtractive manufacturing techniques, materials property and processing often need to be optimized simultaneously, and this can be automated by machine learning. For example, the development of new sensors will require optimizing sensing properties together with high-efficiency, low-cost manufacturing, especially with hybrid materials. Multi-material and 3D/4D printing technologies will rely more on AI/ML (artificial intelligence/machine learning) methods [132], to revolutionize composition design for tailored viscosity, dynamic responsiveness, curing behavior, thermo-mechanical properties, as well as rapid prototyping-to-production transition.

With digital twins and workflows for materials selection, design, and optimized printing, one can significantly reduce time and costs for future electronics and 3D printing development [133]. For instance, composites with up to 91% filler were formulated with an elastomer matrix for 3D printing using an image-driven ML model and finite element analysis (FEA) [134]. Interfacial properties in 3D printed particle-matrix composite can be modeled with ML, FEA, and empirical experiments for composite sensor design [135]. ML and structure-composition-processing property (SCPP) relationship are combined to 3D print polyvinylidene fluoride (PVDF)/molybdenum disulfide (MoS_2_) composites with boosted piezoelectric sensing behavior, providing a promising route to the design and fabrication of high-performance flexible electronics and wearable device via AI-aided 3D printing [132,136].

In addition, templated or guided assembly is widely used to fabricate functional nanomaterials [137,138,139], organic electronics [140], and ionics [141,142]. These methods offer unique self-assembly-induced properties in a vast processing parameter space (including varied molecular structures of the crystallization mediator and assembly conditions) and can be combined with 3D printing to more efficiently optimize the resultant nanostructures and electrical performances.

### 4.5. Levels of Automation

Three-dimensional-printed wearable electronics and biosensors are showing promise in recent developments [143,144,145,146,147,148,149,150,151]. We expect that the application of the above-mentioned AI algorithms will further automate the design and popularization of these devices, ranging from glucose sensors, sweat sensors, strain and tactile sensors, oximeters, artificial skin, electroencephalography (EEG), and electrocardiography (ECG) sensors, temperature sensors, to energy storage, interconnects, and energy harvest devices [143,144,145,146,147,148,149,150,151]. We expect that automated, AI-based (3D) electronics printing will continue to thrive and benefit the revolution of Industry 4.0 [152,153,154,155,156,157,158,159,160].

**Figure 11:**
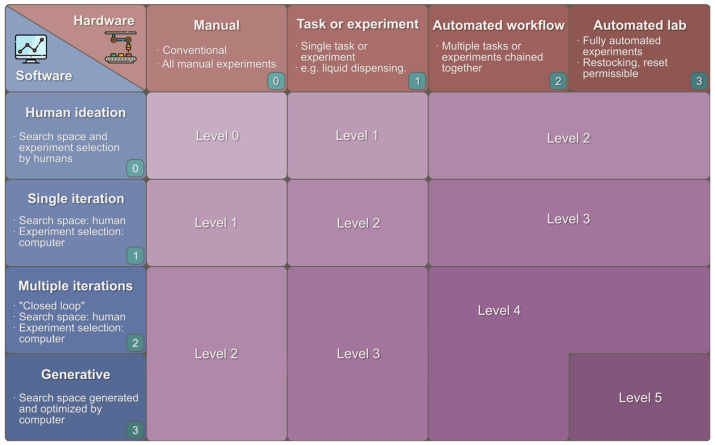
Schematic for the autonomy levels of SDLs based on the category of hardware and software autonomy achieved. (Figures and Captions above are from reference [170] without change, under Creative Commons Attribution 4.0 International License: https://creativecommons.org/licenses/by-nc-nd/4.0/, accessed on 15 May 2025) SDL refers to Self-Driving Laboratory.

AI automations can range from different levels of a so-called self-driving lab or SDL (Figure 11). Currently, most reports are at level 1 or 2. For example, AI-enhanced 3D printing can explore the vast printing design space efficiently, use printing parameters as features to predict product properties, achieve optimal sensors or devices with minimal experimental trial-and-errors, as well as efficient ink selection, performance prediction, enhanced modeling, and printing optimization [152,153,154,155,161,162,163,164,165,166,167,168,169,170].

### 4.6. Possibilities

**Figure 12:**
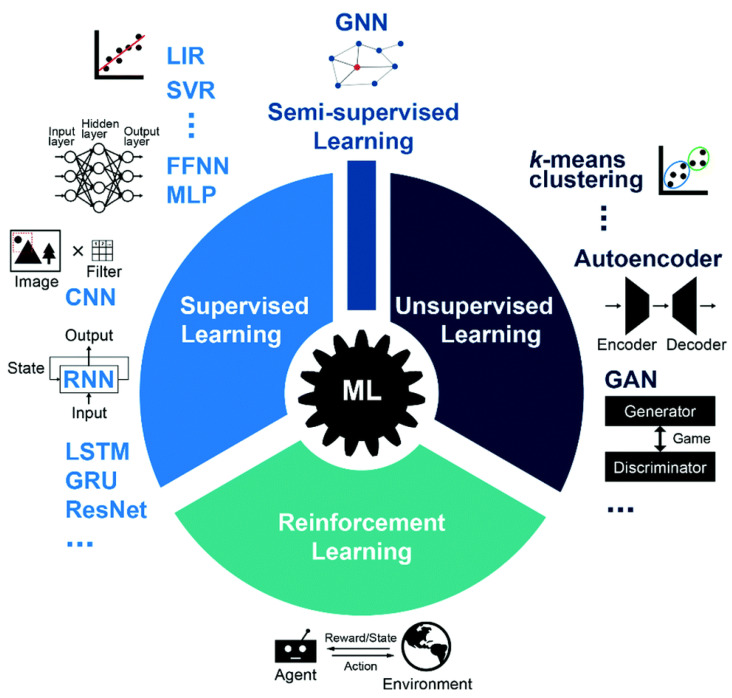
A brief overview of ML approaches, including three major categories known as supervised learning, unsupervised learning, and reinforcement learning. ML approaches such as linear regression (LIR), support vector regression (SVR), feedforward neural networks (FFNNs), multilayer perceptron (MLP), convolutional neural networks (CNNs), and recurrent neural networks (RNNs) are generally used for supervised learning. Typical approaches to unsupervised learning include k-means clustering and autoencoder and generative adversarial networks (GANs). Reinforcement learning follows a general interactive loop between the agent and the environment. The difference between supervised and unsupervised learning is determined by whether training data is labeled or unlabeled, and there is a category of tasks between them called semi-supervised learning, which combines labeled and unlabeled data (generally mostly unlabeled) during training. It is worth pointing out that some of the aforementioned ML methods are not merely limited to the tasks illustrated in this schematic. For instance, graph neural networks (GNNs) have been widely used for semi-supervised learning tasks, but they are also applicable to supervised and unsupervised learning tasks involving graph representation. (Figures and Captions above are from reference [161] without change, under Creative Commons Attribution 4.0 International License [75].).

Figure 12 gives a general classification of AI and machine learning models [161], which consist of unsupervised and supervised learning, semi-supervised learning, as well as reinforcement learning. Unsupervised learning is a class of data-driven techniques without human inputs or labeled data, whereas supervised learning is trained with labeled data (or ground truth) [3,40,118]. Reinforcement learning relies on the interactions between agents and their surroundings [3,40,118]. The effectiveness of supervised and unsupervised learning is monitored with a loss function, while reinforcement learning maximizes a reward sum. In addition, semi-supervised learning utilizes training from data with and without labels. Other ML techniques include Linear regression (LIR in Figure 11), support vector regression (SVR, or support vector machine), random forest, and gradient boost, while deep learning covers convolutional neural networks (CNNs), feedforward neural networks (FFNNs), multilayer perceptron (MLP), long short-term memory (LSTM), recurrent neural networks (RNNs), gated recurrent unit (GRU), generative adversarial network (GAN), ResNet or Residual Network, and variational autoencoder (VAE) [3,40,118].

**Figure 13:**
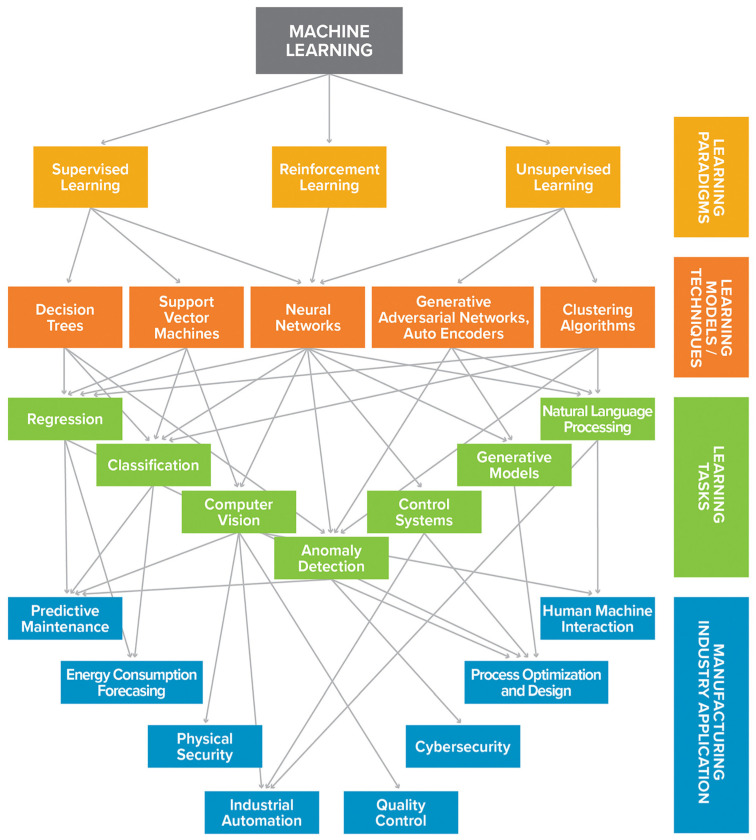
Common categories for various aspects of machine learning, grouped into paradigms, techniques, tasks, and relevant manufacturing industry applications. (Figures and Captions above are from reference [162] without change, under Creative Commons Attribution 4.0 International License [10]).

Figure 13 shows important areas of AI implementation in manufacturing, including 3D printing. Supervised learning, unsupervised learning, as well as reinforcement, are promising in various downstream tasks, including preventative maintenance, predictive consumption, automation, quality assurance, optimization, human-machine interface, and cyber security [162]. Table 3 summarizes some of the recent important applications of AI in electronics printing.

## 5. The Future of AI-Aided Electronics Printing

### 5.1. Recent Examples: Soft Robotics and Wearable Electronics

**Figure 14:**
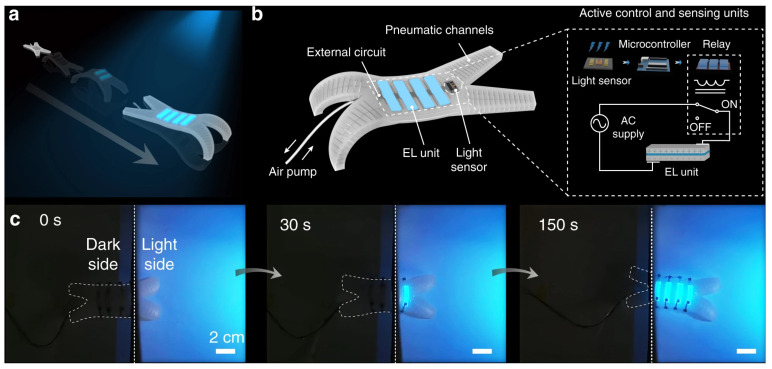
Control logic and the spatially color-changing ability of the ELbot. (**a**) Schematic illustrating the color-matching strategy of the ELbot in imitation of the color-changing ability of chameleons. (**b**) Control logic of the ELbot. (**c**) Instantaneous color-changing ability of the ELbot. The corresponding EL devices on the soft robot were instantly illuminated when they were exposed to a blue light. Scale bar: 2 cm. (Figures and Captions above are from reference [150] without change, under Creative Commons Attribution 4.0 International License [10]).

**Figure 15:**
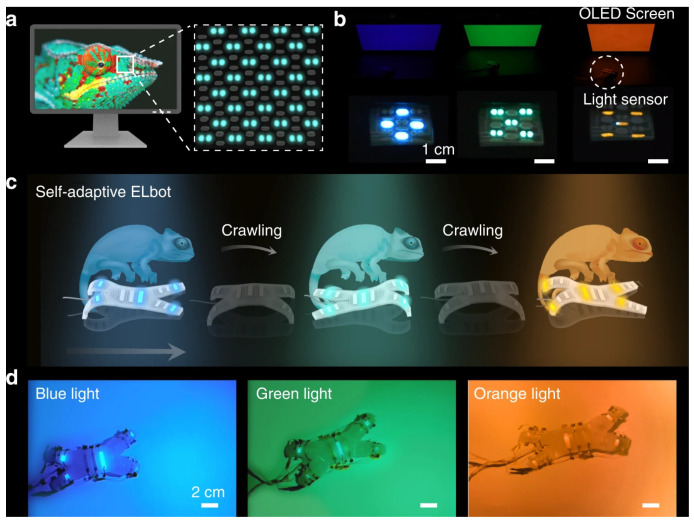
(**a**) Schematic illustration of a self-adaptive color-matching EL display. The chameleon picture is adapted from Pixabay under the CC0 Creative Commons License. (**b**) Instantaneous color-changing of the EL-integrated display in response to the background light variation. Blue, green, and orange light-emitting EL units were printed on the display. A 200-V AC power voltage at a frequency of 1000 Hz was set. Scale bar: 1 cm. (**c**) Schematic illustrating the chameleon-inspired background-matching strategy of the ELbot. The chameleon sketches are adapted from Freepick under the CC0 Creative Commons License. (**d**) Instantaneous color-changing of the ELbot in response to the background light variation. Scale bar: 2 cm. (Figures and Captions above are from reference [150] without change, under Creative Commons Attribution 4.0 International License [10]).

**Figure 16:**
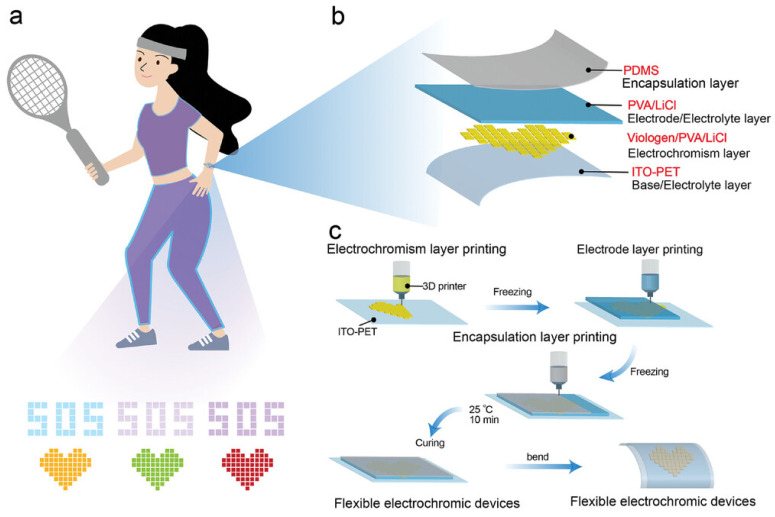
Integrated 3D printing of hydrogels-based FECDs (**a**) The heart-shaped array and SOS-type array are made of ETV hydrogel and STV hydrogel, respectively. (**b**) Structural design of integrated 3D printing of hydrogel-based FECDs. FECDs consist of a four-layer stacked structure, which includes an encapsulation layer (PDMS), an electrolyte layer/electrode layer (PVA/LiCl hydrogel), an electrolyte layer (viologen/PVA/LiCl hydrogel), and a base/electrode layer (ITO-PET). (**c**) Integrated 3D printing processes of FECDs. The array of electrochromic layers is printed in three consecutive steps via a multi-material printing nozzle. (Figures and Captions above are from reference [143] without change, under Creative Commons Attribution 4.0 International License [10]) FECD refers to flexible electrochromic devices.

Three-dimensional-printed electronics are currently tested or adopted in areas such as energy storage, medical devices, wearable electronics, and soft robotics. Figure 14, Figure 15 and Figure 16 highlight two of the examples. Figure 14 and Figure 15 show a 3D-printed soft electroluminescent robot (ELbot) that relies on the background-adaptive electroluminescent (EL) to imitate chameleons. Figure 16 has a 3D-printed example of wearable electrochromic devices (FECDs), which are developed as a wristband to display vital signals in real time. The FECDs in Figure 16 have four layers: DIW-printed Polydimethylsiloxane (PDMS) encapsulation, DIW-printed polyvinyl alcohol (PVA)—LiCl hydrogel electrode, DIW-printed conjugated-molecule, viologen- based hydrogel electrolyte, and flexible Indium-Tin-Oxide (ITO) coated Polyethylene terephthalate (PET) electrode as substrate.

### 5.2. High Throughput Experimentations

AI applications have both advantages and limitations in 3D printing and Electronics printing. The advantages include scalability, automation, data-driven discovery, and potentially out-of-box, unconventional insights. Along with explainability, computational cost, and the necessity of human guidance, the most critical limitations may include the current availability of high-quality data and the time-consuming process of digital twin building.

**Figure 17:**
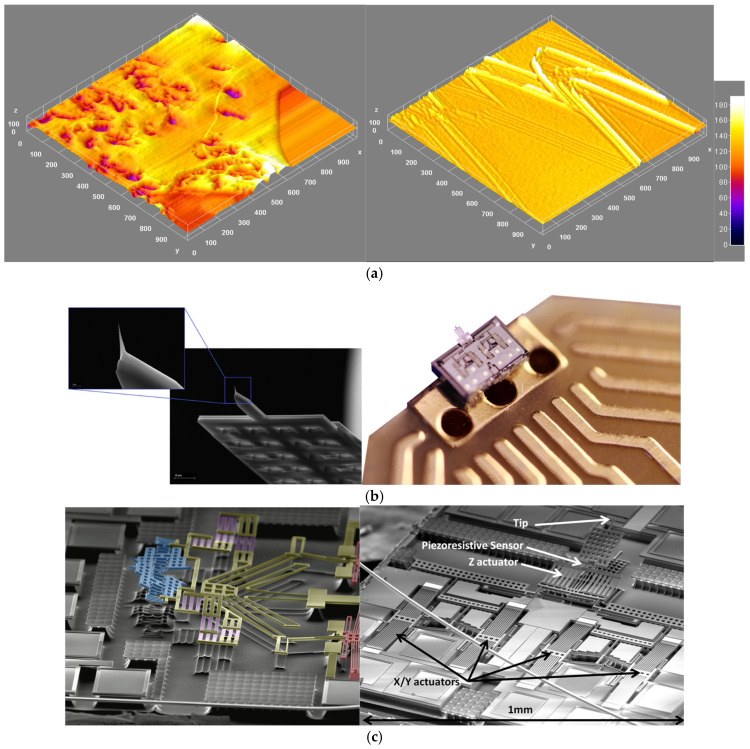
(**a**) Automated Atomic Force Microscopy (AFM) of a paper battery by Redux AFM from ICSPI. High throughput imaging is enabled via microelectromechanical-systems (MEMS)-based AFM design. (**Left**): a 3D phase image of an anode made of graphite, glycerol, and carboxylmethyl cellulose (CMC) on a 25-micron-pore filter paper from Ahlstrom-Munksjo filtration LLC (Scan size 10 by 10 micron); (**Right**): a 3D phase image of a cathode made of MnO_2_, CMC, graphite on the same filter paper. (Scan size 17 by 17 microns) (**b**) Diagrams and micrographs of MEMS-based AFM devices manufactured using a Complementary Metal-Oxide-Semiconductor (CMOS) process. (**Left**): Close-up SEM micrograph of a diamond-like carbon (DLC) tip with radius <20 nm located at the end of a cantilever. (**Right**): Optical micrograph of a MEMS AFM device (1.275 mm × 0.8 mm × 0.275 mm) mounted onto the end of a carrier Printed Circuit Board (PCB). (**c**) SEM micrograph of MEMS-based AFM devices. (**Left**): Colorized SEM micrograph of a MEMS AFM device showing XYZ electrothermal actuators and integrated piezoresistive sensor, allowing for self-sensing and eliminating the need for laser alignment. (**Right**): Locations of sensors and actuators (Images provided by ICSPI, https://www.icspicorp.com/, 15 May 2025).

Figure 17 provides an example of tackling data availability with high throughput and automated experimentation [111]. Legacy AFMs use miniature silicon probes, complex laser-based detection systems, and piezoelectric stages and thus require multiple manual alignment steps. While advances have been made to AFM instrumentation to achieve higher resolution, faster scan speeds, and more exotic modes, ease of use and time to data have not been priorities. The AFM-on-a-chip technology simplifies AFM operation in a significant way (Figure 15), using Micro-Electro-Mechanical Systems (MEMS) technology to incorporate scanning and sensing onto a single chip [171]. Each MEMS chip is capable of extremely precise movement in XYZ for scanning, and each chip has built-in sensors for nanoscale measurement and the automatic approach. This leads to much reduced time to data, greater ease of use, and the ability to generate significantly greater quantities of AFM data. In addition to the benefits described above, there are several technical benefits of using MEMS over legacy optical- and piezo-based AFM, such as lower drift, greater vibration immunity, and no hysteresis or creep (a common problem in AFM due to the piezoelectric scanners).

**Figure 18:**
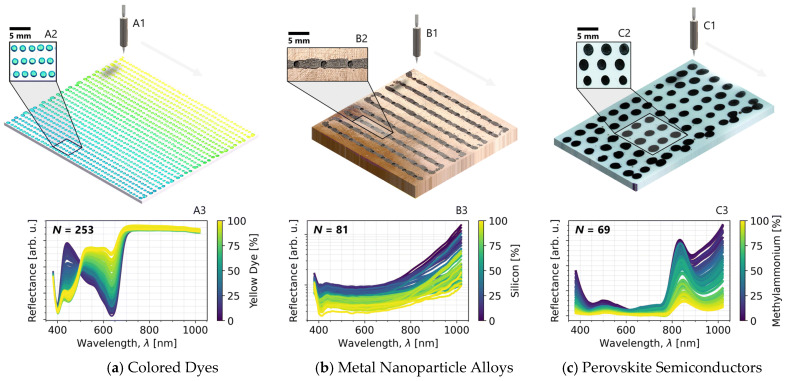
General high-throughput combinatorial printing use-cases of Archerfish. Demonstrated deposition and optical characterization of (**a**) blue-to-yellow colored dye, (**b**) titanium–silicon metal nanoparticle alloys, and (**c**) formamidnium lead iodide–methylammonium lead iodide (FAPbI3–MAPbI3) hybrid organic–inorganic perovskite semiconductors. The Archerfish print head follows a tool path to print (**A1**) a gradient of 253 closely packed droplets onto a hydrophobic coated polyester sheet (100 mm × 100 mm, PP2500, 3M), (**B1**) 20 layers of continuous gradient line segments lines onto a sanded copper plate (80 mm × 80 mm) with 3 mm diameter hemispherical wells, and (**C1**) a gradient of 69 loosely packed, large droplets onto a glass slide (50 mm × 75 mm). Magnified views of the (**A2**) dye droplets, (**B2**) metal alloys, and (**C2**) perovskite semiconductors. Hyperspectral reflectances of all printed samples for the (**A3**) dye droplets, (**B3**) metal alloys, and (**C3**) perovskite semiconductors. (Figure and caption are from reference [172], without changes, under CC BY NC 3.0 licensee [173]).

Three-dimensional printing itself can also enable high throughput combinatorial testing (HTCT) via in-situ mixing or diffusion [172]. Figure 18 demonstrates a 3D printing HTCT setup for colored dyes, metal nanoparticles, and perovskite semiconductors discovery, with the ability to efficiently explore the vast experimental parameter space available [172].

### 5.3. Environmental Considerations

Printed electronics can be a concern for environmental protection, as it easily blurs the line between plastics recycling and electronics recycling. However, if more biodegradable polymers and environmentally friendly processes are used, it will facilitate our goals of a sustainable future. In addition, the smaller footprint in printed electronics or 3D printing personalized electronics can be beneficial for environmental protection as compared to traditional factories.

Artificial intelligence and machine learning algorithms are increasingly playing a pivotal role in promoting sustainable practices of 3D printing and electronics printing, particularly through the optimization of biodegradable polymer selection, energy-efficient curing processes, and overall low-energy fabrication workflows. By leveraging machine learning, researchers can predict material properties and biodegradability profiles of novel bio-based polymers, thereby streamlining the identification of environmentally friendly alternatives to traditional plastics [119,174]. In photopolymer-based AM, AI techniques have the potential to optimize visible-light-induced photopolymerization—a lower energy alternative to UV curing—by fine-tuning reagent chemistry, exposure time, initiator concentration, and layer thickness, which enhances polymerization efficiency while minimizing energy consumption and reduce health hazard [175,176,177,178].

### 5.4. Conclusions

Printing electronics and AI are a natural combination of our progress with the Internet of Things, Industrial Revolution 4.0, and smart manufacturing. The continued interactions between the two will spark inspiration for a personalized, environment-friendly, and energy-efficient future for Mankind. This review summarizes recent advances in electronics printing (especially with additive manufacturing), and AI-aided electronics printing, laying a foundation to inspire future developments in machine learning and algorithm-driven manufacturing of flexible electronics, supercapacitors, batteries, sensors, and fuel cells with reduced cost and higher performance. A number of recent works overviewed the current status of printed electronics [1,4,20] and 3D printed electronics [3,4,40,41,44–[49]. Goh, Sing, and Yeong also reviewed AI and machine learning-assisted 3D printing [3]. As far as we know, this current work represents the first systematic effort to summarize the recent trends in AI-assisted electronics printing, especially 3D electronics printing.

To summarize, the following perspective points highlight the findings and discussions in this review:

1. Traditional techniques of electronics printing provide contact and non-contact pattern transferring to fabricate flexible electronics with low-cost, low-temperature processes.
2. Additive manufacturing of electronics offers opportunities for multi-material, multi-process, personalized fabrication of sensors, electronics, energy storage, and harvest devices, which are not easily accomplishable by traditional ways of electronics printing.
3. AI and algorithm-based optimization vastly improve the efficiency of 3D printing and electronics printing via a high-quality database, digital twin building, and process optimization.

We are currently moving from Level 1 and 2 automation of self-driving labs towards Levels 3 and 4. It may be extremely costly and risky to completely remove a human scientist from the loop. Nevertheless, the future of AI-assisted electronics printing and 3D printing lies in formulating new materials for inks or filaments, optimizing high throughput processes, automating new end-application scenarios, and developing efficient algorithms.

Despite promising results, several challenges must be addressed to fully realize AI-enabled electronics printing. First, the availability of large, labeled datasets remains a bottleneck, especially for rare defect classes or novel materials. Second, the explainability of AI models—particularly deep learning architectures—remains limited, which raises concerns for safety-critical applications like biomedical sensors. Third, the transferability of trained models across different printing systems, inks, and substrates is not guaranteed, necessitating further research into domain adaptation and physics-informed AI models. Nevertheless, ongoing advances in edge computing, in-situ sensing, and hybrid modeling approaches continue to strengthen the integration path of AI in electronics printing technologies.

## Figures and Tables

**Table 1:** Comparison of 3D printing technologies [50,51,52,53,54,55,56].

Method	Accuracy (mm)	Material Compatibility	Surface Finish	Print Speed	Strength	Limitation	Equipment Cost
FDM	±0.05 –0.3	Thermoplastic	Moderate	0.04–0.15 m/s	Low cost, Robust	Layer lines	$200– $15,000
SLA	±0.025 –0.1	Photopolymer	Excellent	10–100 mm/h	High accuracy	Limited materials	$2500– $25,000
DLP	±0.025 –0.1	Photopolymer	Excellent	20–100 mm/h	Faster than SLA	Limited materials	$5000– $50,000
SLS	±0.05 –0.2	Thermoplastics & composites	Rough	10–50 mm^3^/s	Mechanical properties	Powder hazard	$30,000– $200,000
Poly Jet	±0.01 –0.085	Photopolymer	Excellent	0.1–0.2 m/s	Very High Resolution	Expensive	$50,000– $500,000
DIW	±0.1 –0.5	Exceptionally wide	Moderate	0.001–0.5 m/s	Print multi- materials	Viscosity requirement	$10,000– $100,000

**Table 2:** Comparison of AI Models in 3D printing applications [119,120,121,122,123,124,125,126,127,128,129].

Method	Accuracy (%)	Interpret -Ability	Training Need	Use Cases	Adaptability to New Uses	Computational Cost/Complexity
SVM	80–85	High (SHAP)	Low– Moderate	Defect classification, Parameter prediction, Optimization	Moderate	Low to Moderate
ANN	80–97	Low	High	Mechanical property prediction, thermal modeling	Limited	Moderate to High
CNN	92–98	Low– Moderate	High	Layer inspection, defect detection from images	Limited	High
GAN	90–99	Low	Very High	Generation of synthetic data, design	Limited	Very High
Random Forest	80–90	Moderate (feature importance)	Moderate	Materials Screening	High	Moderate
BO	N/A (Optimi- zation)	Moderate (Visualizable)	Low	Multi-parameter tuning	High	Moderate
Pareto Front	Optimi- zation	Low	Moderate	Multi-parameter trade-off	Moderate	High

**Table 3:** Recent works on AI-assisted electronics printing.

Improvements by AI	Materials/Process	Model	Ref
Enhanced interfaces and sensor design	3D printed particle-matrix composite	Computer vision, finite element analysis	[135]
Enhanced reliability	3D printed neuromorphic circuits	Gradient-based optimization	[158]
Jetting prediction for new inks	Inkjet printed electronics	Decision trees, random forest, gradient boosting, and neural networks	[156]
Estimating electromagnetic characteristics	Spiral antennas	Gaussian regression	[157]
Property estimation	Printed circuit board (PCB)	Computer vision for automatic optical inspection (AOI)	[159]
Resistivity modeling of printed lines	3D aerosol-jet printing	Convolutional neural networks	[164]
Predicting resistance	PCB	Computer vision	[165]
Relating print parameters to physical and electrical properties	Inkjet-printed electronics	Nonlinear regression, k-nearest neighbor (KNN), Gaussian process regression	[166]
Ink selection system	Screen-printed electronics	MLP	[167]
Identification of optical quality and functionality	Inkjet-printed electronics	Computer Vision	[163]
Feature classification	Evaporation-drivenmulti-scale 3D printing	SVM	[168]
Classification of electrical conductivity based on voltage, nozzle speed, and flow rate	Electro-hydrodynamic-jet of organic electronics	Decision tree classifier, KNN, random forest	[169]
Strain and humidity sensorsfor health monitoring	Graphene-carbon nanotube ink for jet printing	SVM	[154]
